# Advancing teleoperation for legged manipulation with wearable motion capture

**DOI:** 10.3389/frobt.2024.1430842

**Published:** 2024-12-11

**Authors:** Chengxu Zhou, Yuhui Wan, Christopher Peers, Andromachi Maria Delfaki, Dimitrios Kanoulas

**Affiliations:** ^1^ Intelligent Robotics Research Group, Department of Computer Science, University College London, London, United Kingdom; ^2^ School of Mechanical Engineering, University of Leeds, Leeds, United Kingdom

**Keywords:** teleoperation, legged robots, mobile manipulation, whole-body control, human-robot interaction, telexistence

## Abstract

The sanctity of human life mandates the replacement of individuals with robotic systems in the execution of hazardous tasks. Explosive Ordnance Disposal (EOD), a field fraught with mortal danger, stands at the forefront of this transition. In this study, we explore the potential of robotic telepresence as a safeguard for human operatives, drawing on the robust capabilities demonstrated by legged manipulators in diverse operational contexts. The challenge of autonomy in such precarious domains underscores the advantages of teleoperation—a harmonious blend of human intuition and robotic execution. Herein, we introduce a cost-effective telepresence and teleoperation system employing a legged manipulator, which combines a quadruped robot, an integrated manipulative arm, and RGB-D sensory capabilities. Our innovative approach tackles the intricate challenge of whole-body control for a quadrupedal manipulator. The core of our system is an IMU-based motion capture suit, enabling intuitive teleoperation, augmented by immersive visual telepresence via a VR headset. We have empirically validated our integrated system through rigorous real-world applications, focusing on loco-manipulation tasks that necessitate comprehensive robot control and enhanced visual telepresence for EOD operations.

## 1 Introduction

Within the realms of defense and security, there exist numerous tasks that carry inherent risks too substantial for human operators. Between 2015 and 2020 the United Kingdom saw around 2,000 Explosive Ordnance Disposal (EOD) operations annually, a testament to the substantial need for safeguarding human life and expertise in such operations. The level of human expertise required for these operations is notably high. Consequently, to safeguard human life and welfare, the development of robotic substitutes is vigorously pursued by defense departments globally.

Robotic platforms designed for navigating difficult terrains and handling objects are particularly suited for these high-risk tasks. To the best of our knowledge, this study is the first to use legged robots to complete an EOD task. Recent advancements have seen legged quadrupedal robots become more affordable and robust when operating in unstructured environments, such as uneven ground and staircases. These robots now possess advanced computational capabilities, including GPUs, and are equipped with an extensive array of sensors, such as Inertial Measurement Units (IMUs), cameras, and force/torque sensors. Notably, the RGB-D sensors offer depth perception critical for EOD tasks, enabling the robot to understand the 3D structure of environments (especially the wires). A pioneering step in this field is the integration of robotic arms onto quadruped robots, endowing them with enhanced loco-manipulation abilities. Notable examples of such platforms include Spot by Boston Dynamics, ANYmal by ANYbotics, as well as HyQ and CENTAURO developed by the Italian Institute of Technology. A legged quadruped manipulator is deemed exemplary for executing complex loco-manipulation tasks.

Despite significant focus within the robotics community on autonomous scene comprehension and action execution, replicating human-like skill proficiency remains a formidable challenge. High-level decision-making for intricate tasks continues to be an intense area of research. Therefore, managing the high-level actions of robots through teleoperation and telexistence represents a pragmatic intermediate towards achieving full autonomy. Robot teleoperation and telexistence is a field with established roots Mason (2012), recently resurgent in research interest, stimulated by substantial advancements in computational units, sensor technology, and wireless communication, which are now potentially capable of supporting robust real-time performance.

**FIGURE 1:**
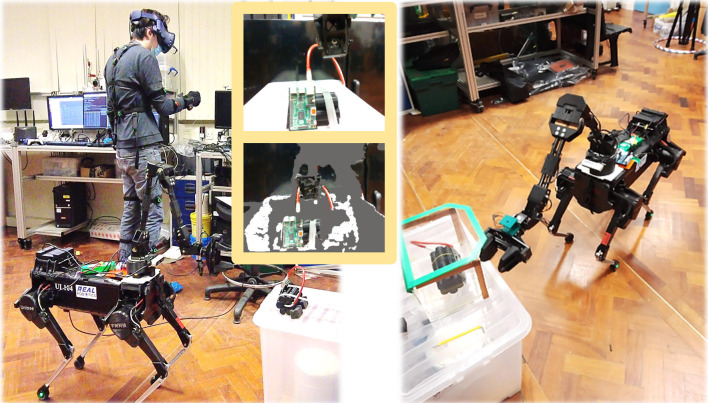
Teleoperative legged manipulator system: a teleoperator with VR headset and wearable IMU-based motion capture, controlling locomotion and manipulation.

In this paper, we first detail the development of a cost-effective yet capable quadrupedal manipulator (Figure 1). The system comprises a Unitree Laikago quadruped robot, a re-engineered lightweight 5 Degrees-of-Freedom (DoF) ViperX 300 robotic arm to conserve the robot’s payload capacity, and an Intel Realsense D435 RGB-D camera, which offers real-time depth maps to support tasks like bomb identification and manipulation. Subsequently, we introduce a comprehensive system for integrated whole-body loco-manipulation control. This is facilitated through the use of a wearable IMU-based motion capture system for teleoperation (Perception Neuron Motion Capture), communicating via 5 GHz Wi-Fi six for real-time interaction. With this system, an operator can intuitively control both the robot’s locomotion and manipulation tasks simultaneously, receiving visual feedback through a depth camera relayed to a Virtual Reality (VR) headset (HTC Vive Pro). The HTC Vive Pro headset, known for its high-resolution display and wide field of view, enhances operator immersion and situational awareness. Our aim is to enable straightforward, real-time robotic teleoperation and telexistence for the execution of perilous defence and security tasks, exemplified by EOD operations. To validate the system’s efficacy, we conducted real-world experiments focused on EOD scenarios.

The principal contributions of this paper are threefold:

1. We introduce a novel approach to the hardware assembly of an economical quadrupedal manipulator, which includes the adaptation of the robotic arm into a more lightweight version to conserve the robot’s carrying capacity.
2. We present a new, unified teleoperation system for loco-manipulation, employing a wearable IMU-based motion capture system—a technology not previously utilised in quadrupedal legged robotics research.
3. Through real-world experimentation, we demonstrate the superior performance of our telexistence and teleoperation system in completing EOD tasks, in comparison to conventional gamepad-based methods.

The paper is organised as follows: Section 2 reviews related work in the field of robot teleoperation and telexistence. The description of the hardware system is provided in Section 3. Section 4 delineates the whole-body teleoperation system, while Section 5 details the empirical validation of the system using a physical robot. We conclude with a discussion of our findings and suggest avenues for future research in Section 6.

## 2 Related work

Traditionally, joystick control served as the primary method for teleoperating legged and wheeled robots Chestnutt et al. (2006); Klamt et al. (2018). However, more recent research has explored mixed-reality (MR) and virtual reality (VR) approaches, which provide immersive and intuitive control over complex systems. Cruz Ulloa et al. have developed mixed-reality tele-operation methods for high-level control of legged-manipulator robots, showcasing the potential of such systems for enhanced user experience and precision Cruz Ulloa et al. (2022). Additionally, their work analyzed the effectiveness of MR–VR teleoperation methods, offering insight into the strengths of both approaches in managing the complexities of legged-manipulator robots Cruz Ulloa et al. (2024).

Wearable devices have also emerged as a promising control interface for teleoperation. For example, the MULT (Mechanical Upper Limbs Tracker) system, designed for teleoperation, exemplifies a highly accurate, wearable system that tracks human arm movements for robot control. Palagi et al. demonstrated the capabilities of this system for efficient teleoperation tasks, emphasizing its utility in managing legged robots through wearable technology Palagi et al. (2024).

Although sufficient for the basic manoeuvring of a robot’s body on level surfaces, joysticks present limitations when complex movements are required, such as the 6DoF motion of a manipulator’s end-effector, which can be more effectively managed using motion capture hand controllers Zhang et al. (2018). The challenge compounds when the simultaneous control of multiple robot components is necessary—for example, coordinating the position/velocity of a robot’s body with that of its end-effector. This complexity has been evident in our work with the IIT-Centauro robot Rolley-Parnell et al. (2018) and observed in studies on the ETH-ANYmal’s legged manipulation Bellicoso et al. (2019). Consequently, the research domain has shifted focus to explore various devices and sensors for more complex legged manipulator teleoperation.

Wearable exoskeletons represent one such alternative, offering a more immersive experience for the operator. These systems, despite their cost, have proven effective for manipulation tasks Klamt et al. (2020). However, separating navigational control—often managed by a joystick—from manipulation has introduced an undesirable level of complexity. Alternative approaches to locomotion control, such as treadmills, have been proposed Elobaid et al. (2019), though they come with significant financial implications.

Motion capture technology, widely used for manipulation control, encounters its own set of limitations. For instance, Deshpande et al. (2018) combined motion capture with a haptic device for arm and hand control to teleoperate a quadrupedal manipulator, yet locomotion control remained unaddressed, often pre-set prior to manipulation tasks. Additionally, the stationary installation requirement of most motion capture setups hinders the mobility of the teleoperator, who ideally would have the freedom to move in various environments.

Our work takes inspiration from the animation industry and employs an IMU-based wearable suit for robot teleoperation, where the IMU system measures the motion of the human body and transforms the measured orientations into movement commands for a robot. Some of the research uses the upper body motion of the human only to control robot arms Škulj et al. (2021); Yang et al. (2016a). Prior research indicates that IMU-embedded wearable motion capture devices can effectively generate both high and low-level control signals for robots, enhancing precision in complex tasks. Examples of more complex systems include the control of a bipedal robot through high-level walking motions Kim et al. (2009) and a wheeled robot through gesture-based commands using an IMU device on the hand and arm Shin et al. (2014). These systems have also shown their reliability and accuracy for controlling 7 DoF robotic arms Yang et al. (2016b) and in domestic settings, such as assistance for individuals with dementia Lv et al. (2020). Some research extends the IMU-based teleoperation to mobile manipulators Wu et al. (2019); Hirao et al. (2023), which gives the system even more DoFs. In Dalin et al. (2021), a motion tracking suit facilitates the control of the TALOS bipedal robot as a static system, utilizing a human model to adapt the suit data to the robot’s capabilities. Our previous work designed an interface for human operators to control a quadrupedal in simulation Peers et al. (2021). While previous works have explored elements of IMU-based control, our study introduces a novel approach that leverages an IMU suit for the integrated control of navigation and manipulation specifically in a quadrupedal legged manipulator operating in real-world environments. This dual-function application is, to our knowledge, unprecedented in the field and represents a unique advance by enabling simultaneous control of both locomotion and task-oriented manipulation.

In the realm of telexistence, current methodologies predominantly incorporate VR headsets to provide visual feedback Elobaid et al. (2019), supplemented occasionally by haptic devices to enhance the operator’s control and perception Tachi et al. (2013). Our approach aligns with these vision-based methods, using VR to afford the operator with telexistence capabilities, while deferring the integration of haptic feedback to future endeavors.

## 3 Hardware system description

**FIGURE 2:**
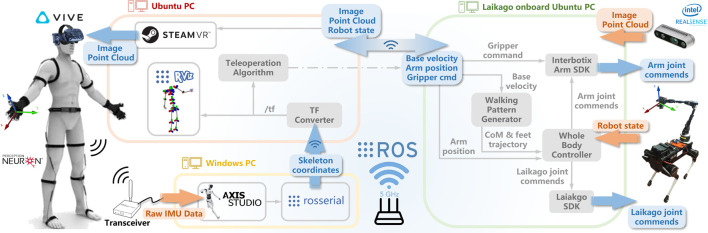
System overview: the teleoperator (left) controlling the robot base and arm (right), via wearable IMU-based motion capture, while getting feedback from the robot’s RGB-D camera via the VR headset.

This section delineates the hardware configuration employed for robotic teleoperation and telexistence, along with modifications implemented to enhance functionality. An overarching schematic of the system is depicted in Figure 2, comprising 1) an integrated robotic apparatus, which includes a quadruped robot with an affixed robotic arm and gripper, alongside an RGB-D sensor, and 2) a human teleoperation setup, featuring a VR headset and an IMU-based motion capture suit.

The robotic hardware bifurcates into two components. The foundational element is the quadruped base, typified by the Unitree Laikago, capable of a 5 kg payload and energized by an onboard Intel NUC i5 processor. The robotic appendage, a ViperX 300 robotic arm, crowned with an Intel RealSense RGB-D camera, constitutes the secondary component. The arm’s design underwent a re-engineering process to mount seamlessly atop the quadruped chassis. Additionally, the camera is designed to pivot along the yaw axis in synchrony with the arm. A comprehensive simulation of the robot was crafted within the Robot Operating System (ROS) and Gazebo platforms to verify operational competencies prior to real-world deployment.

Teleoperation is facilitated by the Noitom Perception Neuron Studio, an inertia-driven wearable motion capture system. It encompasses 16 IMU sensors, strategically placed on human body joints, and Studio Motion Capture Gloves for detailed finger tracking. This assemblage offers high-fidelity tracking of the human operator’s skeletal posture and limb orientations globally, which include 19 body segments and 40 hand segments, delivering real-time updates at 100 Hz with a minimal resolution threshold of 0.02°. A swift calibration process, requiring roughly 30 s, is prerequisite for each novel user.

The visual immersion is provided via an HTC VIVE Pro VR headset, portraying the robotic entity, the human operator, and sensor-derived data like point clouds and RGB imagery from the robot’s vision system. To ensure latency-free interaction within dynamic environments, communication across all devices is maintained through a 5 GHz Wi-Fi six network.

### 3.1 Hardware redesign

**FIGURE 3:**
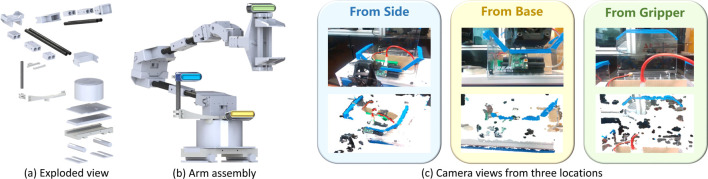
**(A)** Exploded view of all redesigned components in the robotic arm, **(B)** full assembly of the robotic arm with three possible camera mounting positions colored and **(C)** the corresponding RGB and depth camera image from each mounting location on the robotic arm.

Addressing the payload limitation inherent to quadruped robot platforms, we have adopted various strategies to minimize the additional load on the Laikago base. The primary concern was the 5-DoF ViperX 300 robotic arm, which represented the heaviest component of our design. Our redesign efforts focused on preserving the arm’s functionality while reducing its weight. We replaced the aluminum links with carbon fiber rods and re-engineered the shoulder joints to accommodate the new materials. These modifications achieved a significant reduction in the arm’s weight from 4.1 kg to 2.3 kg, amounting to a 44% decrease. The lighter arm preserves the Laikago base’s stability and maneuverability, allowing for additional manipulative tasks. We integrated the arm onto the Laikago using a 3D-printed slide-in mounting system affixed to the rear carbon fiber rods, designed for quick and easy interchangeability. A fully rendered illustration of the redesigned arm and the Laikago mounting system can be viewed in Figure 3.

For visual feedback, an RGB-D Intel RealSense camera was employed to facilitate telexistence. This setup not only provides RGB imagery critical for navigation and manipulation tasks but also enhances the operator’s spatial awareness with colored depth-based point clouds. The latter is particularly valuable for intricate tasks requiring precise distance measurements, like opening containers or severing wires. The optimal camera placement was determined through extensive validation tests, considering various positions and angles to enhance the teleoperation experience. The camera was ultimately positioned on the robot arm, ensuring a consistent perspective irrespective of the arm’s yaw motion. We evaluated three distinct mounting locations: (1) at the base of the arm with a slight upward pitch, (2) on the wrist joint behind the end-effector, and (3) laterally offset at a calibrated height with a minor yaw rotation towards the arm. Renderings depicting these configurations and their custom 3D-printed mounts are presented in Figure 3.

In practice, we observed that the arm’s position occasionally obscured the end-effector from the operator’s view, complicating precise tasks. This challenge was notably prevalent when the end-effector was required to interact with objects vertically. By positioning the camera laterally, akin to a “third-person” perspective, we mitigated this issue, significantly enhancing the operator’s ability to gauge the end-effector’s positioning in both horizontal and vertical planes. This strategic camera placement ensured a clear view of both the manipulated object and the end-effector without obstruction. We also noted that the point cloud’s fidelity was compromised when the camera was mounted too close to the subject, leading to unreadable points. This insight informed our final decision on the camera’s location, optimizing both visibility and point cloud quality for effective teleoperation.

## 4 Software system description

**FIGURE 4:**
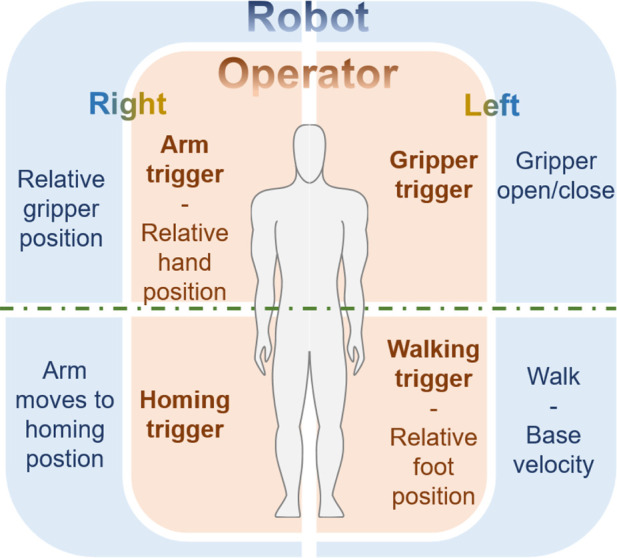
IMU-suit.

After detailing the hardware adjustments, the focus shifts to the software architecture underpinning the teleoperation and telexistence capabilities. This software system is comprised of three primary modules: robotic motion control, teleoperation facilitated by IMU feedback (Figure 4), and the generation of a VR environment for telexistence.

### 4.1 Gait generation and whole-body control

The gait generation for the quadruped robot leverages the concept of virtual legs Sutherland and Ullner (1984)—a strategy inspired by bipedal robotics, where pairs of legs are coordinated to move as one. This concept allows for the adaptation of existing bipedal walking algorithms to quadrupedal motion patterns, such as trotting, pacing, and bounding. By considering diagonal pairs of legs as ‘virtual legs’, we have extended a bipedal walking algorithm Zhou et al. (2017) to produce a quadrupedal trotting gait. The trajectory planning, depicted in Figure 2, feeds into a whole-body control framework that orchestrates the locomotion and manipulation tasks of the robot.

The whole-body controller that considers the full dynamics of the developed legged manipulator is formulated as a quadratic programming problem as described in Equation 1:

$$\underset{X}{\min}\;\overset{n}{\underset{i=1}{\sum }}\omega_{i}||A_{i}X-b_{i}|{|}^{2},$$ (1)

where the sum of n task’s costs is minimized to obtain the optimal value of the target variable, $X={\left[\ddot{q},\lambda \right]}^{T}$ , which consists of the generalized acceleration $\ddot{q}$ and contact wrenches λ . The $i^{\text{th}}$ task is defined by an objective matrix and vector, $A_{i}$ and $b_{i}$ , and a weight $\omega_{i}$ that determines the soft priorities between tasks. For an in-depth understanding of the tasks and their specific formulations, one can refer to the literature You et al. (2016).

During the optimisation process, the following constraints are considered: i) floating base dynamics $M_{f}\ddot{q}+h_{f}=J_{f}^{T}\lambda$ , ii) joint torque limits $M_{a}\ddot{q}+h_{a}-J_{a}^{T}\lambda =\tau \in \left[\tau_{\text{min}},\tau_{\text{max}}\right]$ , iii) non-slip contact constraints $J\ddot{q}+\dot{J}\dot{q}=0$ , and iv) contact force constraints to keep each contact force within a linearised friction cone. M is the inertia matrix, h is the sum of Coriolis, centrifugal and gravitational terms, J is the contact Jacobian matrix corresponding to the contact wrenches λ at all contact points, the subscript f represents the top six rows of a matrix for the floating base and a corresponds to the actuated DoFs, $q={\left[q_{f},q_{a}\right]}^{T}$ are the generalised coordinates which consists of the 6 DoF floating-base coordinates $q_{f}$ , and the actuated joints $q_{a}$ , τ is a vector of joint torques.

The optimized result X is then used to calculate the joint torque, joint position, and joint velocity references for lower-level control of the developed legged manipulator You et al. (2016).

### 4.2 Teleoperation strategies

**FIGURE 5:**
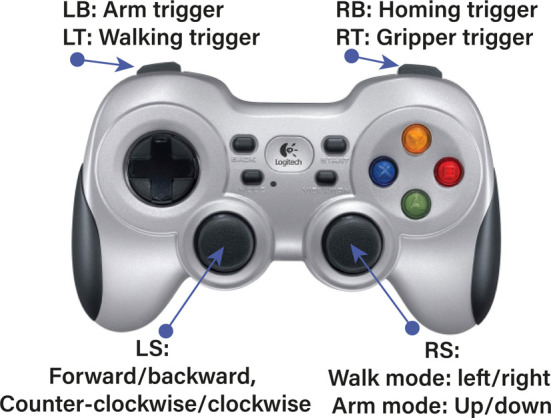
Gamepad.

The capture of human body motion is executed by a wearable motion capture system, specifically the Noitom Perception Neuron, as depicted in Figure 2-left. This system delivers stable and precise estimations of human body segment poses. The accompanying software, Noitom Axis Studio, is capable of capturing comprehensive skeletal data, including nuanced finger movements. This data is subsequently transmitted to ROS via the *rosserial* protocol. Given the inherent kinematic disparities between the human operator and the robot, a direct joint-level linkage is unfeasible. Accordingly, we have engineered four intuitive strategies for the teleoperation of the legged manipulator to conduct locomotion and manipulation tasks. As illustrated in Figures 4 and 5, hand closure by the teleoperator serves as a command trigger to the robot.

• *Gripper trigger* engages when the teleoperator’s left hand closes above waist level, causing the manipulator’s gripper to remain shut. This grip is maintained until the trigger is disengaged.
• *Walking trigger* is initiated when the left hand closes below the waist. The teleoperator can then relay base velocity references to the walking pattern generator by moving in a desired direction or turning. The magnitude of the robot’s velocity corresponds to the extent of the teleoperator’s step or turning angle. The robot halts upon the release of this trigger.
• *Arm trigger* commences when the right hand is closed above the waist, translating the teleoperator’s right arm movements into relative Cartesian position references for the robot’s arm via the whole body controller. This motion ceases when the trigger is disengaged.
• *Homing trigger* is activated by closing the right hand below the waist, which moves the robot’s joints to a pre-set configuration.

The triggers for the left and right hand closures can be simultaneously engaged, allowing for concurrent locomotion and manipulation control of the legged manipulator by using the left hand to enable the *walking trigger* and the right for the *arm trigger*.

The movement relationship between the teleoperator and the robot is scaled relatively, with the *arm* and *walking triggers* at time *t* modeled as described in Equation 2:

$$\left\{\begin{array}{cc} a_{rd}^{t}=a_{rd}^{0}+\mu \left(a_{e}^{t}-a_{e}^{0}\right)\; & \text{arm trigger}\\ {\dot{w}}_{rd}^{t}={\dot{w}}_{rd}^{0}+\mu \left(w_{e}^{t}-w_{e}^{0}\right)\; & \text{walking trigger} \end{array}\right.$$ (2)

where $a=\left[x,z,\theta_{\text{roll}},\theta_{\text{pitch}},\theta_{\text{yaw}}\right]$ and $w=\left[x,y,\theta_{\text{yaw}}\right]$ represent displacements and rotations in the sagittal (x) , lateral (y) , and vertical (z) planes, and rotations about the longitudinal $\left(\theta_{\text{roll}}\right)$ , lateral $\left(\theta_{\text{pitch}}\right)$ , and vertical $\left(\theta_{\text{yaw}}\right)$ axes for the arm and base movements respectively. The subscript *e* denotes the teleoperator, *r* the robot, and *d* the desired value, with the superscript *0* indicating the initial instance of trigger activation. The scaling factor μ is employed to adjust the teleoperator’s motions to suit the robot’s scale. Hand closure, which is detected by the VR glove’s sensors, activates the triggers that teleoperate the robot.

Employing a trigger-based relative motion strategy effectively circumvents the prevalent sensor drift issue, especially with the yaw angle—a common detriment to controllers dependent on absolute yaw detection.

### 4.3 VR-based integration for telexistence and teleoperation

In a myriad of practical scenarios, maintaining a direct visual link between the robot and the teleoperator is not feasible. Operations such as those conducted in confined spaces, involving hazardous materials (HAZMAT), or EOD necessitate that the teleoperator is stationed at a secure location, often out of sight from the robot. As a result, an alternative remote monitoring system becomes indispensable to manage the robot’s movements and to comprehend its environment from a distance. Moreover, given that the wearable motion capture system hinges on translating the teleoperator’s bodily movements into control signals, it is impractical for the operator to view a stationary display while physically rotating or moving. Virtual Reality (VR) technology not only overcomes this limitation by providing an immersive full-view experience but also enhances the operator’s telexistence, offering a more comprehensive and interactive environment than conventional displays, thereby facilitating robust and effective robot control. During the experiments, the teleoperator’s perception is confined exclusively to the input from the VR headset, ensuring the integrity of the teleoperative experience.

The VR environment is constructed by integrating the perspectives of both the robot’s and the teleoperator’s states, enriched with visual RGB-D feedback. Initially, the robot’s RGB-D camera supplies the teleoperator with a colour-infused 3D point cloud alongside a 2D RGB image superimposed above the cloud representation (as depicted in Figure 3C). The RGB image offers an immediate view of the surroundings, while the point cloud conveys a more intricate depiction of the environment. Notably, the colored images are compressed prior to transmission to enhance data transfer efficiency. The point clouds are confined to a 2-m radius to provide a focused and prompt rendering of the vicinity. We have examined various point cloud filtration methods, including downsampling techniques like voxelisation and statistical outlier removal. Achieving a balance between precision and succinct representation is challenging; finer elements such as wires are represented by minimal points within the cloud, which leads to their exclusion when significant filtering is applied.

Subsequently, both the robot’s digital model and the teleoperator’s IMU-derived skeletal representation are rendered within the VR environment. This dual visualization enables the teleoperators to observe not only the robot and the visual landscape surrounding it but also their own virtualised body structure, culminating in a comprehensive telexistence experience. Data transmission between the robot and the VR headset is carried out via 5 GHz Wi-Fi, selected for its capacity to handle the necessary bandwidth. Preliminary experiments, including VR-assisted manipulation, were conducted using a mobile manipulator within the nascent stages of the UCL MPPL development Liu et al. (2020).

## 5 Experimental results

Gamepads are prevalent in robot control systems, with many quadruped manufacturers supplying them as the standard control device for their commercial clients. In our study, we aimed to assess the efficacy of the wearable motion capture system in contrast with a conventional gamepad (Figure 5). To ensure a fair comparison, both control methods employed identical teleoperation strategies: velocity control for locomotion and position control for manipulation. Specifically, the gamepad’s trigger and bumper buttons were programmed to correspond to triggers in the teleoperation system, and the joysticks were configured to regulate relative position and velocity. Participants were asked to teleoperate a legged manipulator robot to complete a series of tasks using both types of controllers, with the completion time of each task serving as the performance metric.

### 5.1 Experimental design

**FIGURE 6:**
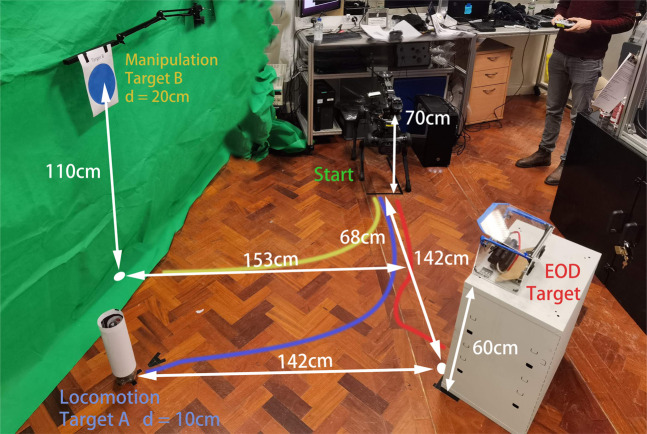
Detailed map of the experiment area with the locations of each target.

The experimental protocol consisted of four distinct tasks, depicted in Figure 6. Participants commenced with an orientation task to familiarize themselves with the teleoperation controls using both the gamepad and the motion capture system. Following this, they were required to perform a locomotion task, a manipulation task, a combined loco-manipulation task, and an EOD task. To mitigate sequence effects, the order of the locomotion and manipulation tasks was randomized: half of the participants conducted the locomotion task first, while the other half started with the manipulation task. Subsequently, all participants undertook the EOD task under consistent conditions, devoid of direct visual contact, relying solely on VR for visual input.

1. In the locomotion task, participants directed the robot from a starting position to target A.
2. The manipulation task involved initiating from a position beneath target B, with the objective of maneuvering the robotic arm from its home pose to reach target B, which was suspended in the air.
3. For the loco-manipulation task, participants navigated the robot from the starting point towards target B and then operated its arm to reach target B aloft.
4. The final EOD task simulated a practical application scenario where the robot had to approach a bomb placed inside a container, starting from the initial point. The robot was then required to open the container and extract a red wire from the bomb using its manipulator.

Criteria for task failure included the robot veering outside the designated experimental boundary, improper or damaging movements of the robotic arm towards the box, or any technical malfunction within the control system, robot, or arm that led to operational incapacity.

### 5.2 User profiles

Total of 12 (4 female, eight male) participants’ data was collected for the study. Among these, 11 participants completed all the experiment tasks within a reasonable time frame without failure, and user No. Six was unable to complete the combined task due to tactical challenges. These participants’ ages ranged from 21 to 32 years old (mean age: 25.4 years), and they reported an average computer gamepad experience of 2.5 (on a scale of 0–9). The volunteers received basic instructions on how to use the controllers but were not offered additional practice time before commencing the tasks.

### 5.3 Gamepad vs. IMU suit performance comparison

**FIGURE 7:**
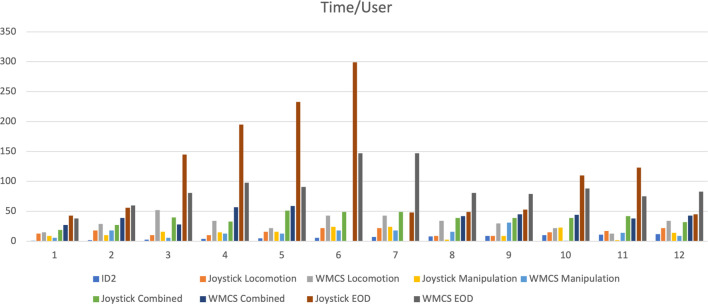
Movement time each user takes to complete tasks with gamepad (GP) and IMU-based wearable motion capture system (WMCS).

The completion times for each experimental stage by all twelve participants are presented in Figure 7. Table 1 provides the average completion times using different control interfaces. It is observed that the gamepad yields better performance in simple locomotion tasks, whereas the wearable motion capture system exhibits an edge in more complex operations. Moreover, there is a noticeable improvement in user performance with the motion capture system as the experiment progresses.

**TABLE 1:** Average time to complete each task and its standard deviation in seconds.

	Gamepad		IMU		IMU + VR
	Avg	Stdev	Avg	Stdev	Avg
Locomotion	14.5	4.5	31.2	12.7	
Manipulation	14.8	5.2	12.3	4.8	
Loco + Mani	17.5 + 17.6	12.2	33.2 + 8.2	14.3	
EOD	160.1	102.3	83.6	38.1	115

Statistical analysis through paired t-tests confirms these observations quantitatively. When considering all tasks, the analysis shows no significant difference in overall completion times between the interfaces ( t=0.976 , p=0.341 ). However, focusing on the EOD tasks, a statistically significant advantage is observed for the motion capture system over the gamepad, with completion times significantly faster for the motion capture system ( t=2.796 , p=0.038 ). This supports the system’s efficacy in complex and critical operations where efficiency is paramount.

In the locomotion task, the completion time with the wearable motion capture system was nearly double that of the gamepad. For manipulation tasks, the times are comparably close, with less than a 20% variance. Here, the gamepad demonstrates superior efficacy in straightforward locomotion tasks.

For tasks combining locomotion and manipulation, the overall completion times for both control methods are closely matched. However, a disaggregated analysis into the locomotion and manipulation components reveals that the gamepad ensures an even time distribution between the two (locomotion at 17.5 s and manipulation at 17.6 s). In contrast, the motion capture system experiences difficulties with the locomotion component but outperforms in manipulation (locomotion at 33.2 s and manipulation at 8.2 s). This discrepancy may be attributed to the differing Degrees of Freedom involved: locomotion being a 2D movement and manipulation a 3D one, rendering the latter more complex in our setup. Gamepad joysticks typically afford 2-DoF control, well-suited for 2D movements, whereas the motion capture system’s 3D spatial tracking aligns naturally with the manipulator’s 3D operations. Consequently, for human teleoperators, controlling a robotic arm via arm movements is both intuitive and user-friendly, conferring an advantage on the motion capture system in applications requiring complex manipulations.

An illustrative case is the EOD task, which demands both locomotion and intricate manipulation, with the latter posing greater complexity. As indicated in Table 1, the gamepad required nearly twice as much time to complete the EOD mission as the wearable motion capture system, on average. In missions where a prompt response is critical to prevent damage, injury, or loss of life, the heightened efficiency of the motion capture system proves invaluable for teleoperating legged manipulators in real-world scenarios.

**FIGURE 8:**
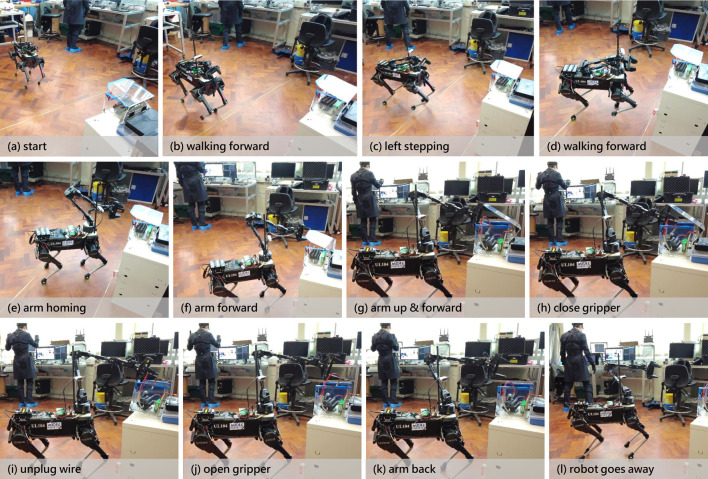
Chronological snapshots of the teleoperator’s command with the corresponding teleoperated system’s action throughout the live experiment.

The experimental procedure, progressing from simpler to more challenging tasks, facilitates user familiarization with the system. Analysis of Table 1 reveals a performance enhancement trend with the wearable motion capture system, notably a greater than 30% improvement in the manipulation segment of the combined task compared to the stand-alone manipulation task earlier. This trend was not observed with the gamepad. Moreover, the performance disparity between the control methods widened with additional practice, favoring the motion capture system. Post-training, one user was able to complete the EOD task in less than 45 s with less than 2 hours of additional practice, halving the initial time. Figure 8 provides snapshots from a training session, reinforcing the potential for improvement with the motion capture system.

The manipulation tasks targeted by this system necessitate precision to the centimeter level. Our experiments with EOD tasks necessitated approximately 1 cm movement accuracy, thereby affirming the efficacy of the proposed IMU-based teleoperation system.

### 5.4 VR usability

Throughout the experiment, all participants were able to accomplish the EOD task using the wearable motion capture system in conjunction with VR integration. According to Table 1, the task was completed in an average time of 118 s. This duration was longer than when using the motion capture system with direct line-of-sight but was still more efficient than utilising the gamepad with direct sight.

The camera was mounted adjacent to the robot’s manipulator to provide a detailed view of the object being manipulated. Participants reported that while this setup offered enhanced detail during manipulation tasks, the camera’s limited field of view proved challenging during locomotion phases due to its narrow angle.

Despite this limitation, no participants reported discomfort as a result of using the VR system during the tests. A majority of the users endorsed the use of VR as a viable option for operations in environments that are not accessible to humans, suggesting an overall positive reception for the VR component of the system.

## 6 Conclusion

The integration of quadrupedal legged manipulation with teleoperation stands at the forefront of contemporary robotic research and development, epitomizing a paradigm shift towards augmenting human operational capacity within treacherous or inaccessible environments. Our investigation within this burgeoning field has culminated in the development of a teleoperated system specifically engineered for EOD operations. However, telexistence remains an ambitiously complex endeavor, replete with challenges in delivering a dependable teleoperation or VR system for pragmatic robotic deployment.

One of the paramount challenges we encountered pertained to the harmonization of hardware and software elements. The primary hardware limitation stemmed from the inherent payload restrictions of legged robots; in our case, the maximum payload did not exceed 5 kg. This necessitated the utilization of only lightweight components for the robot’s construction. We addressed this limitation by customizing the design of the manipulator affixed to the robot. On the software side, the primary issue revolved around the synchronization of sub-modules distributed across three distinct computing systems (Figure 2). Complicating this was the necessity to bridge components compatible with different operating systems—Ubuntu for the quadruped, manipulator, and RGB-D sensor, and Windows for the VR and wearable IMU-based motion capture. Our system architecture, alongside the communication facilitated by 5 GHz Wi-Fi, was designed to overcome this complexity. Calibration of the wearable IMU-based motion capture system was expedited, requiring mere seconds, and potential calibration drifts were mitigated through the adoption of intuitive movement adjustments by the teleoperator, ensuring accurate robot control.

During the experiments, operator feedback was confined to visual inputs, either through direct line of sight or VR immersion. The onus of ensuring safe robot-environment interactions rested on the robot’s low-level joint torque controller. A future aspiration is to incorporate haptic feedback, which we anticipate will significantly enhance the Human-Robot Interaction (HRI) experience.

Prospective enhancements to our system are multifold. Firstly, we are exploring the development of software capable of running all devices under a unified operating system, which could potentially reduce the number of necessary computers and diminish synchronization complications. Secondly, our sights are set on semi-autonomous teleoperation, eschewing a strict one-to-one correspondence between teleoperator inputs and robotic actions in favor of a system where the robot assumes autonomous control of certain cognitive tasks, guided by the teleoperator’s strategic oversight. Finally, we intend to extend the application of our system beyond EOD tasks to include a variety of real-world functions, such as industrial inspection and monitoring.

An important observation during our experimental trials was that user proficiency with gamepad controllers did not significantly impact the system’s efficacy. Despite varying levels of gamepad experience, all participants—comprised of college students and researchers—were able to operate the system effectively. Nonetheless, this homogeneity among test subjects does highlight a limitation in our study; a broader demographic might yield varied outcomes. Future studies could benefit from a more diverse participant base to assess the system’s accessibility and performance across a wider spectrum of users.

## Data Availability

The raw data supporting the conclusions of this article will be made available by the authors, without undue reservation.
